# SOX Family Members Expression in Canine Oral Melanoma: Role of the SOX3 in Tumor Aggressiveness

**DOI:** 10.3390/vetsci12090851

**Published:** 2025-09-02

**Authors:** Renato Felipe Costa, Bárbara Andrade de Carvalho, Bruna Mendes Lima, Emerson Soares Veloso, Karen Yumi Ribeiro Nakagaki, Ivy Nayra Nascimento Gonçalves, Helen Lima Del Puerto, Enio Ferreira

**Affiliations:** Department of General Pathology, Institute of Biological Sciences, Universidade Federal de Minas Gerais, Belo Horizonte 31270-901, Brazil; natofelipe16@gmail.com (R.F.C.); babiandrade13@gmail.com (B.A.d.C.); brunamendeslima@gmail.com (B.M.L.); emerson.esv@hotmail.com (E.S.V.); karenyumi@ymail.com (K.Y.R.N.); ivynayra489@gmail.com (I.N.N.G.); helendelpuerto@hotmail.com (H.L.D.P.)

**Keywords:** SOX, immunohistochemistry, melanoma oral, canine

## Abstract

The aggressiveness of oral melanoma can be related to several mutations that occur during development. Based on the knowledge of the role of transcription factors of the SOX family in other neoplastic types, it is necessary to understand their behavior in oral melanomas. In this work, the expression of SOX2, SOX3, and SOX10 and its relationship with the proliferative index and histological aspects indicative of aggressiveness in canine oral melanomas were evaluated. Thirty tumors were histologically reviewed and the expression of Melan-A, SOX2, SOX3, SOX10, and Ki67 in these tumors were determined. All tumors presented histomorphological characteristics compatible with malignant tumors and immunopositivity for Melan-A. The expression of SOX2, SOX3, and SOX10 was observed in 7/30 (23.3%), 6/30 (20%), and 23/30 (76.6%) of the cases, respectively. Among the analyzed markers, the relationship between the loss of SOX3 expression in tumors with higher proliferative rates was highlighted. An inverse correlation was also observed between the expression cytoplasmic SOX10 and nuclear SOX10, suggesting a change in the location of this protein in oral melanomas. Among the SOX family proteins studied, the SOX3 protein plays a role in the regulation of cell proliferation in oral melanomas, and it is suggested that the SOX2 and SOX10 proteins are constitutively expressed in these neoplasms, without a determining role for aggressiveness. New studies of this gene transcription pathway may assist in possible prognostic and predictive determinations of the SOX3 protein in oral canine melanoma.

## Simple Summary

The aggressiveness of oral melanoma may be related to several mutations that occur during development. Based on the knowledge of the role of SOX family transcription factors in other neoplastic types, it is necessary to understand their behavior in oral melanomas. In this study, the expression of SOX2, SOX3 and SOX10 and their relationship with the proliferative index and histological aspects indicative of aggressiveness in canine oral melanomas were evaluated. Thirty tumors were reviewed histologically and the expression of Melan-A, SOX2, SOX3, SOX10 and Ki67 in these tumors was determined. All tumors presented histomorphological characteristics compatible with malignant tumors and immunopositivity for Melan-A. Among the markers analyzed, the relationship between the loss of SOX3 expression in tumors with higher proliferative rates stood out. An inverse correlation was also observed between the expression of cytoplasmic SOX10 and nuclear SOX10, suggesting an alteration in the localization of this protein in oral melanomas. Among the SOX family proteins studied, the SOX3 protein plays an important role in the regulation of cell proliferation in oral melanomas. New studies of this gene transcription pathway may aid in possible prognostic and predictive determinations of the SOX3 protein in canine oral melanoma.

## 1. Introduction

Oral melanoma is a malignant neoplasm that arises from the proliferation rampage of melanocytes, melanin-producing cells distributed throughout the entire lamina propria of the oral mucosa. Oral melanoma in humans is a very rare neoplasm in the oral cavity and its etiology remains uncertain [1,2]. Despite presenting low incidence in humans, oral melanomas have a high lethality rate, due to their high propensity to metastasis, with approximately 79% of patients dying within five years after diagnosis [3,4].

There are many models of animal studies for oral melanoma, and the canine model has been commonly mentioned [5]. Melanomas in this species share similarity to human neoplasia, such as clinical behavior, therapy responses, and molecular and histopathological characteristics [6].

In dogs, about 62% of melanomas can be seen in the oral cavity, 1% in the eye cavity, 27% in the skin, and 10% in digits [5]. Mortality is attributed to the high metastatic index, with about 58% to 74% of metastases in regional lymph nodes and 14% to 67% in the lungs at the time of diagnosis [7]. Dogs are usually treated with combined surgery, chemotherapy, or radiotherapy [8,9].

When oral melanomas are treated exclusively by surgical excision, approximately 85–90% of cases recur locally or develop distant metastases within 5 to 9 months [10].

The aggressiveness of oral melanoma is related to several mutations involved in its development. Furthermore, melanomas in humans and dogs share mutations in the PI3K and MAPK pathways, which may contribute to the progression of this neoplasia [5]. In this sense, it is suggested that other molecular alterations may be similar between these species, such as the SOX3 protein mutation already observed in human melanomas [11]. Thus, the analysis of SOX3 expression in dogs can help to demonstrate the comparative aspects between these species.

SOX3 is an oncogene in numerous human neoplasms, including osteosarcoma, esophageal carcinoma, glioblastoma, ovarian cancer, and T cell lymphoma [12,13]. In these different neoplasms, the role in controlling the epithelial–mesenchymal transition and increasing cell viability, invasion, proliferation, and migration is reported. Furthermore, its expression has been correlated with a worse prognosis in patients diagnosed with hepatocellular carcinoma [14,15]. However, the pathways of action of SOX3 in canine tissue samples have not yet been studied.

In addition to SOX3, other members of this family have already been studied in tumors. SOX2, a transcription factor belonging to the SOXB1 subgroup of genes, and SOX1 and SOX3, play a vital role in sexual organ embryogenesis, neurogenesis, and oncogenesis [15]. In a complex review study, it was demonstrated that SOX2 is expressed in at least 25 types of cancer and directly implicated in tumor aggressiveness. Detailed analysis of its role in melanocytic neoplasms becomes crucial, since its function is still poorly defined [16]. The expression of SOX2 in human melanomas is directly related to the tumorigenicity and self-renewal of initiator cells and melanoma stem cells [17].

Recent reports demonstrate that SOX2 expression may be present in 50% of melanomas related to neoplastic invasion [18]. However, some authors infer that SOX2 expression is not required for metastasis formation or melanogenesis [19].

Although the expression of SOX2 is quite controversial, and the role of the SOX3 protein has not yet been elucidated, the SOX10 protein, another member of this family of transcription factors, has already been widely studied in melanomas. SOX10 belongs to the SOXE subgroup with SOX8 and SOX9. They play several activities in different cell types, including glial cells, oligodendrocytes, peripheral nervous system cells, and central nervous system, as well as melanocytes [20]. SOX10 is highly expressed in breast neoplasms, hepatocellular carcinoma, salivary cystic adenoid tumors, glioblastoma, and glioma [21]. In melanoma, SOX10 is strongly expressed in primary and metastatic cells, promoting migration and invasion while suppressing melanogenesis and driving senescence [20,22].

Based on the knowledge of the role of transcription factors of the SOX family in other neoplastic types, it is necessary to investigate their expression in oral melanomas. Evaluation of the expression pattern of these proteins may provide relevant prognostic and predictive information in targeted therapy studies. Due to its relationship with aggressiveness in different tumor types, we hypothesized that proteins SOX2, SOX3 and SOX10, in canine melanomas, play a role similar to that observed in human neoplasms.

## 2. Materials and Methods

### 2.1. Ethical Aspects

This work was carried out following the ethical principles for using animals in experimentation and after approval by the Animal Experimentation Ethics Committee (CEUA) of the Federal University of Minas Gerais (Protocol 008/2016).

### 2.2. Cases

Thirty cases of oral melanomas, previously fixed in neutral formalin and 10% buffered and stored in paraffin blocks, from the Cellular Behavior Laboratory file of the Department of Pathology, Federal University of Minas Gerais, were analyzed. The materials were obtained between 2005 and 2019 after surgical excision of dogs treated at different veterinary clinics in Minas Gerais, Brazil.

### 2.3. Histopathological Evaluation

Histological sections of 4 μm of tissues embedded in paraffin, stained with the hematoxylin-eosin (HE) technique, were re-examined to confirm the morphological diagnosis, according to the criteria established for diagnostic confirmation of melanoma [23]. The following characteristics were determined in the histopathological evaluation: the morphology of neoplastic cells (spindle or epithelioid), pigmentation of the lesions, evidence of vascular invasion (neoplastic embolus), perineural invasion, ulceration, desmoplasia, and presence of junctional activity (lentiginous or pagetoid).

### 2.4. Immunohistochemistry

After reviewing the cases, 4 μm histological sections of selected samples were used in the immunohistochemical technique. Samples containing preserved tumor tissue, with minimal areas of necrosis, ulceration, or intense inflammation, were included for analysis. Each tumor was evaluated as a whole, without differentiating between peripheral and central regions. The immunohistoperoxidase method with identification from polymerized secondary antibodies was used in this procedure. Antigen retrieval was carried out using moist heat (bain-marie at 98 °C) for 20 min or pressurized heat at a temperature of 125 °C for 40 min (Pascal^®^), with a citrate solution, pH 6.0 (DakoCytomation Target RetrievalSolution Agilent Technologies, Glostrup, Denmark) or ethylenediaminetetraacetic acid (EDTA), pH 9.0 (DakoCytomation Target Retrieval Solution) diluted to a concentration of 10%, and then cooled to room temperature for 20 min. The slides were incubated in a 3% H202 solution in methyl alcohol to block endogenous peroxidase. The reagents were applied manually, with an incubation time of the primary antibody of 16 h and the other reagents of 30 min, except for the 3,3′-Diaminobenzidine chromogen (DAB substrate system, DakoCytomation), which was incubated for 1 min. The slides were counterstained with Giemsa and Harris Hematoxylin to differentiate melanin pigmentation into green. As positive reaction controls, canine brain tissue was used for SOX2, SOX3, SOX10, and skin for Ki67 and Melan-A. Negative controls were obtained by replacing the primary antibody with a standard mouse, rabbit, and goat serum. Primary antibodies, their sources, clones, and main reagents are shown in Table 1.

### 2.5. Immunohistochemistry Assessment

Immunohistochemical expression was interpreted by a single pathologist (E.F.) with over 20 years of experience. Positivity for Melan-A was identified by cytoplasmic marking of a distinct brownish color in neoplastic cells, assessed by a previously described semiquantitative method: cases that presented cytoplasmic marking in more than 10% of neoplastic cells were considered positive [24]. Nuclear immunostaining in neoplastic cells was considered to determine the proliferative index of the lesions through Ki67 labeling. Identifying hot spots in the tumor was performed, where a total of 500 neoplastic cells per slide, labeled or not, were counted in a 40× objective (Olympus–BX41, Shinjuku, Tokyo, Japan). Within the total number of cells counted, the percentage of the number of cells that presented immunostaining was calculated, thus obtaining the required index. In this frequency analysis, melanomas with high proliferative index (25/30) and low proliferative index (5/30) were separated with the reference cutoff point by the percentage of immune-marked cells (<19.5% and >19.5%) [25]. For the quantification of SOX2, SOX3, and SOX10, ten random histological fields were analyzed under a 40× objective (Olympus–BX41), with each marker assessed according to previously reported methodologies [26,27]. The percentage of SOX2 labeling in neoplastic cells was semi-quantitatively categorized from the percentage of positive cells in the tumor area: 0 (0%), 1 (1–25%), 2 (26–50%), 3 (51–75%), 4 (>75%). Marking intensity was considered visually and stratified as follows: 0 (negative), 1 (low), 2 (moderate), and 3 (high), and the final score was obtained by multiplying the percentage and the intensity score. Thus, for statistical analysis, scores 0 to 4, 6 to 8, and 9 to 12 were considered weak, moderate, and strong, respectively [26]. The percentage of SOX3 labeling in neoplastic cells was categorized semi-quantitatively, based on the rate of positive cells in the tumor area: 0 (0%), 1 (1–10%), 2 (11–50%), 3 (>50%). Marking intensity was evaluated semi-quantitatively and stratified as follows: 0 (negative), 1 (weak), 2 (moderate), and 3 (strong). Finally, an index for SOX3 was determined by multiplying the percentage and labeling intensity scores. Melanomas with a final score below 5 were considered to have low expression of SOX3. Above that, tumors were believed to have high expression of SOX3 [27,28]. The percentage of SOX10 labeling in neoplastic cells was semi-quantitatively categorized based on the rate of positive cells in the tumor area: 1+, 1 to 25%; 2+, 25 to 50%; 3+, 50 to 75% and 4+; ≥75%. Cytoplasmic and nuclear labeling and formation of perinuclear clusters were analyzed [26]. A summary of the analysis of SOX markers is presented in Table 2.

### 2.6. Statistical Analysis

The InStat software (GraphPad, version 3.0), was used for statistical analysis. Data normality was verified using the Kolmogorov–Smirnov test. The histological parameters were categorized as presence or absence, the Ki67 expression was interpreted in absolute numerical terms in correlation tests and categorically (low and high index) in association tests, and the expression of the SOX proteins under study were also categorized numerically according to the score obtained in the immunohistochemical interpretation. To analyze these categorized parameters, the association of variables was evaluated using the Chi-square test, and the correlation using the Spearman test.

## 3. Results

### 3.1. Histopathological Parameters

Regarding the histological subtype, of the 30 cases analyzed, 76.6% (23/30) were epithelioid melanomas, 20% (6/30) were mixed melanomas (presenting cells in epitheloid and fusiform pattern), and only one case, 3.3% (1/30), was fusiform melanoma. The presence of ulcers was observed in 90% (27/30) of the cases. Junctional activity was observed in 73.3% (22/30) of tumors, with pagetoid and lentiginous alterations occurring at comparable frequencies (36.6%; 11/30 each). Desmoplasia was similarly observed in 36.6% (11/30) of cases. The presence of pigmentation and neoplastic emboli was observed in 63.3% (19/30) and 46.6% (14/30) of the cases, respectively. No significant association or correlation was observed between the histopathological findings.

The only case that presented a fusiform pattern presented histological parameters similar to most cases (with the presence of ulcer and junctional activity, pagetoid type, without occurrence of embolus, pigmentation and desmoplasia). An important fact in this case was the high cytoplasmic and nuclear expression for SOX10, as better detailed in the immunohistochemical findings.

### 3.2. Immunohistochemical Evaluation

**Figure 1:**
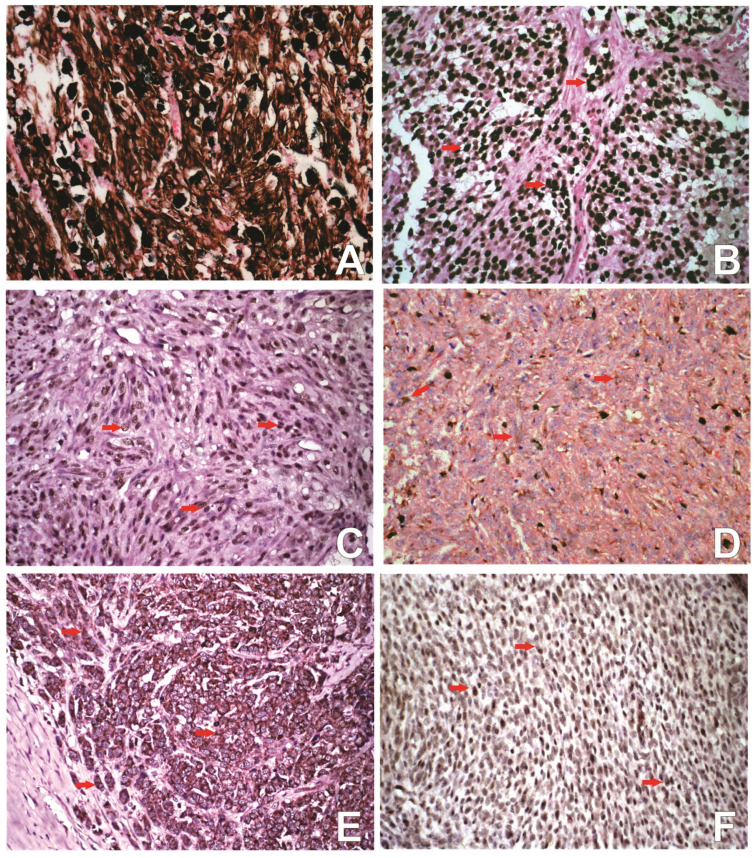
Photomicrograph of oral canine melanoma, counterstaining with Giemsa and Hematoxylin, 400× insert. Image (**A**) Immunostaining for Melan-A showing cytoplasmic positivity in neoplastic melanocytes; (**B**) Immunohistochemical staining for Ki67 demonstrating nuclear labeling in neoplastic cells of a melanoma with high proliferative index (arrows); Image (**C**) Immunostaining for SOX2 in neoplastic cells, nuclear labeling (arrows); Image (**D**) SOX3 immunohistochemistry revealing weak-intensity cytoplasmic immunoreactivity in neoplastic cells, with occasional cells showing moderate staining intensity (arrows); Image (**E**) Immunostaining for SOX10 in neoplastic cells, cytoplasmic labeling (arrows); Image (**F**) Immunostaining for SOX10 in neoplastic cells, nuclear marking (arrows).

The immunoreactivity for melan-A was observed as brown cytoplasmic staining, diffusely distributed in more than 20% neoplastic cells, so all samples were considered positive for this protein (Figure 1A). There was no observable association between the expression of this melanocytic marker and the histopathological parameters analyzed.

For Ki67 expression, a high proliferative index (>19.5) was observed in 86.6% (26/30) of the cases (Figure 1B). In the correlation analysis with the histopathological findings, a significant correlation with emboli formation was observed (*p* > 0.02 and r = 0.48). In the association test, no statistically significant results were observed when comparing melanomas with high and low Ki67 index and the categorical data of the histopathological findings.

The immunostaining of SOX2 protein was visualized in the cytoplasm and nucleus of neoplastic melanocytes, the expression was negative in 76.7% of the cases (23/30), weak in 20% (6/30), and moderate in 3.3% (1/30). No case showed strong expression. In the nucleus, the labeling was strong and heterogeneous in tumor cells, with an expression variation between 5 to 20 cells per high-power field (HPF) (Figure 1C). Cytoplasmic labeling was homogeneous, ranging from weak to moderate in neoplastic melanocytes. The expression of SOX2 did not show any statistically significant correlation with the expression of SOX3, SOX10, Ki-67, and the histological aspects studied.

SOX3 expression was detected in the cytoplasm of neoplastic cells and can also be found in intra- and peri-tumoral lymphocytes. SOX3 expression was observed in 20% (6/30) of the cases; of these, 16.6% (5/30) had a weak expression, and 3.3% (1/30) had moderate expression (Figure 1D). No significant correlation was found between SOX3 expression and the histological aspects studied.

Interestingly, a high expression of Ki67 was observed in tumors with low SOX3 expression (*p* > 0.0052, r = −0.5316). Corroborating the correlation analyses, oral melanomas with positivity for SOX3 were more frequently associated with melanomas with a high proliferative index (*p* < 0.05).

Regarding the SOX10 protein, weak to moderate cytoplasmic immunoexpression was observed in 73.3% (22/30) of the cases studied, and nuclear staining in 20% (6/30) of the issues, with 5 cases scoring 1+ and 1 case scoring 4+ (Figure 1E,F). There was a negative correlation between cytoplasmic SOX10 and nuclear SOX10 expression (r = −0.7948, *p* < 0.0001). However, perinuclear clusters were sometimes observed without correlation with the analyzed parameters. The expression of SOX10 did not correlate with the proteins SOX2, SOX3, SOX10, and Ki67 or with the histopathological characteristics evaluated in this study. Interestingly, the only case that presented a purely fusiform histological pattern showed cytoplasmic expression of SOX10 in most cells (between 50 and 75% of cells) and mainly forming clusters (above 75% of cells).

Detailed case-by-case data on the Ki-67 proliferative index and the immunohistochemical expression scores of SOX2, SOX3, and SOX10 are available in the Supplementary Materials (Supplementary Tables). These include individual tumor scores, classification criteria, and frequency distributions for each marker.

## 4. Discussion

In the present study, we observed that the SOX3 protein indicated greater relevance as an oncogenic marker in the samples of canine oral melanomas evaluated due to its relationship with the proliferative index in these tumors. In contrast, although SOX2 and SOX10 showed positive immunostaining, they did not exhibit statistically significant correlations with histopathological indicators of tumor aggressiveness.

In summary, low SOX3 expression was directly associated with increased cell proliferation in canine melanomas. In human neoplasms, SOX3 expression has been linked to poorer prognosis [27,28,29,30]. In vitro studies in glioblastoma cells have demonstrated that SOX3 is involved in proliferation-related pathways [29]. Additionally, SOX3 localization in the cytoplasm of hepatocellular carcinoma cells suggests it may have functions beyond those typically attributed to a nuclear transcription factor [27].

Its action pathways are still poorly understood in cancer. However, its role in autophagy suppression and activation of the Hedgehog pathway in glioblastomas is already recognized [29]. Furthermore, it is described that SOX3 induces migration and invasion in gastric cancer through the activation of metalloproteinase 926 [30]. SOX3 can be regulated by different transcription factors, including ERK, causing neoplastic cells to play crucial roles in tumor progression, leading the tumor to a more aggressive phenotype [31].

An explanation for the correlation between SOX3 expression and the proliferative index in oral canine melanomas may be based on the fact that SOX3 is associated with cell cycle regulation similar to that observed in osteosarcoma cells [28].

The cytoplasmic expression of SOX3 in oral canine melanomas had not been previously addressed in the literature. Our data indicate that the cytoplasmic expression pattern of SOX3 in oral canine melanomas is similar to other described neoplasms. This alteration in immunoexpression is suggested to be associated with increased proliferation and cellular invasion, potentially leading to reduced survival and a higher risk of tumor recurrence [13]. Further prognostic studies are warranted to better understand this behavior in canine oral melanomas.

It has also been suggested that SOX3 expression may be related to pathways linked to cell differentiation, immune response, NF-Y, and apoptosis, leading tumors with SOX3 expression to a worse prognosis. In addition to these events, SOX3 promotes the suppression of bone morphogenetic protein (BMP) signaling by inhibiting Xvent2 and BMP4 in Xenopus embryos [32]. In breast cancer cell lines, it is suggested that SOX3 expression is related to epithelial–mesenchymal transition, generating implications in tumor biology and contributing to metastatic formation. However, this behavior is not observed in melanomas in the in vitro model A375P [20].

Unlike what was observed for SOX3, the SOX2 and SOX10 proteins did not demonstrate biological significance to the proliferative index or histopathological aspects compatible with the aggressiveness of oral melanomas [23]. The expression of SOX2 in oral canine melanomas may function as a suppressor or activator in alternative signaling pathways, as observed in in vitro studies [12]. Additionally, in hepatocellular carcinomas, gliomas, and colorectal cancers, SOX2 has been shown to induce mesenchymal–epithelial transition and promote invasion [33]. Thus, it is suggested that studies involving the epithelial–mesenchymal transition pathways and invasion in oral canine melanomas may be related to increased SOX2 expression in this tumor type.

Similarly to SOX2 and unlike the literature, which demonstrates the presence of SOX10 in the cell nucleus, our studies indicated the existence of this protein mainly in the cytoplasm, together with the formation of perinuclear clusters in the studied melanomas [2,34]. SOX10 is a protein homogeneously expressed in primary and metastatic melanoma and is essential for melanocyte differentiation. Somatic mutations in SOX10 occur in early-stage melanoma, with SOX10 upregulating MITF, MET, and Nestin expression in melanoma and responding to Wnt signals. Its nuclear localization is controlled by the receptor tyrosine kinase Tam Tyro3 and the transcription factor SOX5 modulates its activity [35]. Overexpression of cytoplasmic and perinuclear SOX10 may indicate its inactive form, mutated, or interacting with other proteins, such as nestin, and therefore, playing a role of greater tumor aggressiveness in oral canine melanomas [36].

One limitation of this study is the variability inherent to immunohistochemical techniques, especially regarding antigen retrieval, which can affect staining quality and reproducibility [37]. Although standardized protocols were applied, the use of anti-human antibodies in canine tissues may have influenced antigen recognition [38]. These factors may explain some inconsistencies observed. Additional limitations include the small sample size and lack of complementary functional assays.

We did not observe any relationship between melanoma aggressiveness, as defined by histopathological features such as vascular emboli, ulceration, and junctional activity, and changes in SOX10 expression. The results obtained in canine oral melanomas were expected to provide innovative data on SOX10 expression and its correlation with the high Ki-67 index. Melanomas with a high proliferative index show high MITF expression; as SOX10 regulates the MITF pathway, it may suggest that invasion in oral melanoma occurs due to low MITF levels and high ZEB1 expression or through direct activation of the activity inhibitory melanoma (MIA) to invasion [18,22]. Therefore, in the samples used, these SOX10 activation pathways need to be studied to investigate a possible correlation between the activation of these pathways and the proliferation of oral melanomas cells.

## 5. Conclusions

Our study demonstrates different expression patterns of three distinct SOX family transcription factors in canine oral melanoma. SOX3 was identified as the most relevant factor in this study, exhibiting a direct relationship with cell proliferation. Regarding transcription factors SOX2 and SOX10, we need to analyze other oncogenic pathways to understand their activities in oral melanoma. SOX regulation in dogs and humans may involve the activation of other tracks in addition to those linked to proliferation, contributing to greater tumor aggressiveness of this neoplastic type. However, its prognostic role must be determined, and functional studies can corroborate the observed results.

## Figures and Tables

**Table 1:** Antibodies and procedures used in the immunohistochemical study.

Antibody	Clone	Manufacturer	Dilution	Antigen Retrieval Method	Secondary Antibody	Incubation Time
SOX2	Polyclonal	R&D Systems	1:100	Citrate + pressurizedheat	Kit ABC *	16 h
SOX3	Polyclonal	Thermo Fisher	1:100	EDTA + wetheat	Anti-Goat **	16 h
SOX10	Polyclonal	Abcam	1:100	Citrate + pressurizedwetheat	Novolink ***	16 h
Melan-A	A103	Dako	1:100	Citrate + pressurizedwetheat	Novolink ***	16 h
Ki67	MIB-1	Dako	1:50	Citrate + pressurizedwetheat	Novolink ***	16 h

* Avidin-Biotin Complex Staining Kits (ABC Kits); Vector Labs’s VECTASTAIN, Burlingame, CA, USA; R&D Systems, Minneapolis, MN, USA; ** Goat anti-Rat IgG (H+L) Secondary Antibody, Biotin; ThermoFisher Scientific, Rockford, IL, USA; *** Novolink Polymer Detection Sistem; Leica Biosystems, Newcastle upon Tyne, UK; Abcam, Cambridge, UK; Dako, Glostrup, Denmark.

**Table 2:** Semi-quantitative assessment of SOX2, SOX3, and SOX10 in melanoma cells.

Marker	Percentage of Positive Cells	Intensity Score	Final Score Calculation	Expression Category
SOX2	0 (0%), 1 (1–25%), 2 (26–50%), 3 (51–75%), 4 (>75%)	0 (negative), 1 (low), 2 (moderate), 3 (high)	% × intensity	0–4: weak; 6–8: moderate; 9–12: strong
SOX3	0 (0%), 1 (1–10%), 2 (11–50%), 3 (>50%)	0 (negative), 1 (weak), 2 (moderate), 3 (strong)	% × intensity	<5: low; ≥5: high
SOX10	1+ (1–25%), 2+ (25–50%), 3+ (50–75%), 4+ (≥75%)	Evaluated visually (cytoplasmic/nuclear, perinuclear clusters)	% × intensity	Not specified

## Data Availability

The original contributions presented in this study are included in the article/Supplementary Materials. Further inquiries can be directed to the corresponding author.

## Supplementary Materials

https://www.mdpi.com/article/10.3390/vetsci12090851/s1, Table S1: Immunohistochemical evaluation of Ki-67 proliferative index and SOX2, SOX3, and SOX10 expression in 30 canine oral melanoma cases; Table S2: SOX2 expression in canine oral melanoma cases; Table S3: SOX3 expression in canine oral melanoma cases; Table S4: SOX10 cytoplasmic and nuclear expression in canine oral melanoma cases

## Abbreviations

The following abbreviations are used in this manuscript:

CEUA	Animal Experimentation Ethics Committee
DAB	3,3′-Diaminobenzidine
EDTA	ethylenediaminetetraacetic acid
HE	Hematoxylin-eosin
HPF	High-power field
MIA	Activity inhibitory melanoma
SOX	SRY-related HMG-box
