# Supplementary material for: SOX Family Members Expression in Canine Oral Melanoma: Role of the SOX3 in Tumor Aggressiveness
Source: Vet Sci. 2025 Sep 2;12(9):851. doi: 10.3390/vetsci12090851 (PMC12474294; doi:10.3390/vetsci12090851)
Supplement: Supplementary file 1 [file vetsci-12-00851-s001.zip › vetsci-3720859-supplementary.pdf]

Table S1. Immunohistochemical evaluation of Ki-67 proliferative index and SOX2, SOX3, and SOX10 expression in 30 canine oral melanoma cases. SOX2 and SOX3 expression levels were scored based on the percentage of positive tumor cells and staining intensity, with final scores categorized as negative, weak, moderate, or strong. SOX10 expression was assessed separately in cytoplasmic and nuclear compartments, with results presented according to semi-quantitative stratification (negative, 1+ to 4+).

CASE	Ki-67 Index	SOX3	SOX2	SOX10 Citoplasmatic	SOX10 Nuclear
1	3.8	0	0	0	0
2	77.6	0	2	4	1
3	53.4	0	0	0	0
4	42.6	0	0	4	0
5	82.8	0	0	4	0
6	32.2	0	8	4	0
7	29.8	1	0	0	0
8	71.8	0	0	4	0
9	55.4	0	0	0	0
10	43.2	0	2	4	0
11	62	0	4	0	0
12	46	0	0	4	0
13	77.6	0	3	0	0
14	58.6	0	0	4	0
15	72.4	0	4	4	0
16	18.1	0	0	4	1
17	17.2	1	0	4	0
18	15.8	1	0	4	0
19	34	0	0	4	0
20	42	0	0	0	0
21	26.5	1	0	2	1
22	7.5	1	0	3	1
23	20.9	0	2	4	0
24	91.7	0	0	4	0
25	91.6	0	0	4	0
26	55.2	4	0	4	0
27	61.6	0	0	4	0
28	77	0	0	4	0
29	49	0	0	2	1
30	27.2	0	0	0	4

Table S2. SOX2 expression in canine oral melanoma cases. SOX2 expression was semi-quantitatively scored based on the percentage of positive tumor cells and staining intensity. Final scores were obtained by multiplying both parameters and classified as follows: scores 0–4 (weak), 6–8 (moderate), and 9–12 (strong). The table summarizes the distribution of cases according to the final classification.

SCORE	SOX2
Negative	23
Weak	6
Moderate	1
Strong	0

Table S3. SOX3 expression in canine oral melanoma cases. SOX3 expression was evaluated semi-quantitatively by combining the percentage of positive cells (0–3 scale) with staining intensity (0–3 scale). The final index was calculated by multiplying both scores. Tumors with a score below 5 were considered to have low SOX3 expression; those above 5 were classified as having high expression.

	SOX3	
Negative	24	
Low	6	
High	0	

Table S4. SOX10 cytoplasmic and nuclear expression in canine oral melanoma cases. SOX10 expression was assessed separately for cytoplasmic and nuclear compartments. Expression levels were semi-quantitatively categorized as follows: 1+ (1–25%), 2+ (26–50%), 3+ (51–75%), and 4+ (≥75% of positive tumor cells). The number of cases per score is indicated for each subcellular compartment.

	SOX10 CITOPLASMATIC	SOX10 NJCLEAR
Negative	8	24
1+	0	5
2+	1	0
3+	1	0
4+	20	1
